# Spatial orientation of social caterpillars is influenced by polarized light

**DOI:** 10.1098/rsbl.2020.0736

**Published:** 2021-02-17

**Authors:** Mizuki Uemura, Andrej Meglič, Myron P. Zalucki, Andrea Battisti, Gregor Belušič

**Affiliations:** ^1^ Department of Agronomy, Food, Natural resources, Animals and Environment, University of Padova, 35020 Legnaro, Padova, Italy; ^2^ Eye Hospital, University Medical Centre, Grablovičeva 46, 1000 Ljubljana, Slovenia; ^3^ School of Biological Sciences, The University of Queensland, St Lucia, Queensland 4072, Australia; ^4^ Department of Biology, Biotechnical faculty, University of Ljubljana, Večna pot 111, 1000 Ljubljana, Slovenia

**Keywords:** larval vision, Lepidoptera, orientation, polarization vision, stemma

## Abstract

Processionary caterpillars of *Thaumetopoea pityocampa* (in Europe) and *Ochrogaster lunifer* (in Australia) (Lepidoptera: Notodontidae) form single files of larvae crawling head-to-tail when moving to feeding and pupation sites. We investigated if the processions are guided by polarization vision. The heading orientation of processions could be manipulated with linear polarizing filters held above the leading caterpillar. Exposure to changes in the angle of polarization around the caterpillars resulted in corresponding changes in heading angles. Anatomical analysis indicated specializations for polarization vision of stemma I in both species. Stemma I has a rhabdom with orthogonal and aligned microvilli, and an opaque and rugged surface, which are optimizations for skylight polarization vision, similar to the dorsal rim of adult insects. Stemmata II-VI have a smooth and shiny surface and lobed rhabdoms with non-orthogonal and non-aligned microvilli; they are thus optimized for general vision with minimal polarization sensitivity. Behavioural and anatomical evidence reveal that polarized light cues are important for larval orientation and can be robustly detected with a simple visual system.

## 1. Background

Movement of animals through the environment is a fundamental part of life history, success, adaptation and evolution. Animals must be able to detect and interpret external cues to navigate to a food and water source, mating ground, shelter and for predator avoidance [1]. External cues include odours, landmarks, celestial bodies, polarized light and magnetic field [2]. The polarized pattern of the sky is used by many insects as a stable spatial reference, which helps them to maintain an orientation and navigate or simply keep a straight line [3,4]. Insects have evolved many adaptations of their visual system for detecting polarized light [4–6].

Extensive research has been done on visual structures that detect polarized light in adult insects from several orders [1]. Compound eyes of most adult insects have a specialized region for detecting skylight polarization pattern, called the dorsal rim area (DRA) [7]. Each ommatidium in the DRA has photoreceptor pairs that sample a common visual angle using orthogonal rhabdomeres with straight and aligned microvilli that are sensitive to two planes of polarization [8]. This arrangement is crucial for achieving high polarization sensitivity (PS) [9]. Polarized light vision has been indicated in larvae of four orders of holometabolous insects (Hymenoptera, Lepidoptera, Trichoptera and Diptera) [10], with behavioural [11–14] and anatomical [15–17] evidence dating 30–70 years ago.

Processionary caterpillars (Lepidoptera: Notodontidae) from Europe and Australia, *Thaumetopoea pityocampa* and *Ochrogaster lunifer*, respectively, have a directed behaviour when mature larvae are ready to pupate [18]. The larvae form a single file crawling head-to-tail and lay down a silk trail when moving to feeding and pupation sites [19,20]. Once the leader of the procession establishes an orientation, the larvae travel along that orientation many metres (10–100 m) per day with minimal deviation [18]. Pheromone trails and physical contact between larvae keep the procession together; however, neither could serve as a guide to a suitable pupation site [19]. So how do these larvae maintain orientation through the environment? We used manipulative behavioural field experiments to determine if processionary caterpillars will react to changes in the angle of light polarization. Morphological analyses of larval stemmata through scanning electron microscopy (SEM), light microscopy (LM) and transmission electron microscopy (TEM) helped identify the likely organ responsible for polarized light vision. Here, we show that the caterpillars strongly react to the presentation of polarized light, and we identify the likely visual organ for its detection, stemma I.

## 2. Materials and methods

### (a) Behavioural analyses

**Figure 1.**
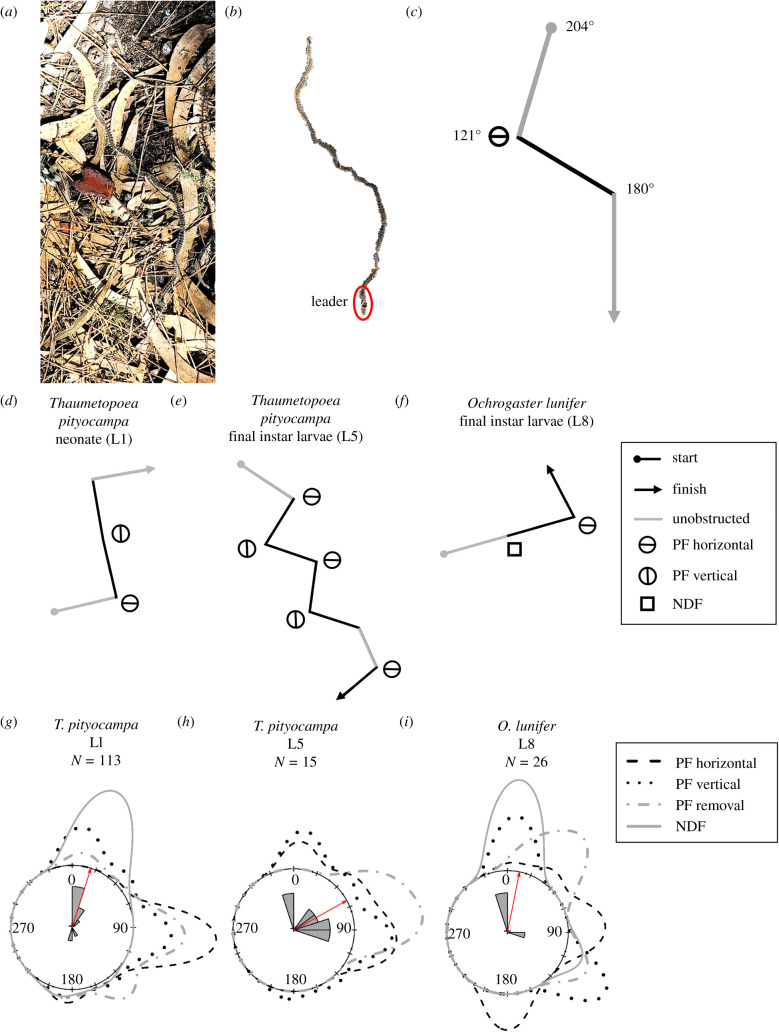
Influence of polarized light on caterpillar heading direction. (*a*) Final instar *T. pityocampa* pre-pupation procession after the experiment with/without a polarizing filter (PF). (*b*) Procession from (*a*) without background. (*c*) Schematic path from (*b*). Numbers indicate the orientation of the procession. (*d–f*) Schematic path of *T. pityocampa* L1, L5 and *O. lunifer* L8, respectively. (*c–f*) Each line segment represents 2 min duration of locomotion. *(g–i*) Circular plots with kernel density estimations (KDE) of summarized angular differences of *T. pityocampa* L1, L5 and *O. lunifer* L8 caused by PF and NDF. The KDE are represented as lines extruding in the outer circle. The rose histograms of *(g,i*) in the middle represent the angular difference after NDF application. In (*h*), NDF was unavailable, therefore data from PF horizontal was plotted. The red arrow represents the mean of the histogram data.

In August 2018 and 2020, outdoor experiments with first instar (L1) *T. pityocampa* larvae were conducted on sunny days between 09.30 and 11.30 h (GMT +2) at Tregnago, Veneto, Italy (45°51′ N, 11°17′ E). A sheet of 50 cm^2^ white paper was used as the experimental arena where 10 first instars at a time were released in the middle of the sheet and behavioural observations were made; *N* = 58 observations (31 single larvae (singletons) and 29 two or more larvae (processions)). After release, the larvae clustered for a few minutes, then formed processions or travelled as a singleton in various orientations. Four treatments were applied to the processions/singletons after the larva(e) established a course. A flexible 25 cm^2^ linear polarizing filter (PF) for visible light (XP42HE-40, ITOS, Mainz, Germany) was bent into a tunnel and held above the procession leader or singleton (electronic supplementary material, figure S1). The PF that filtered the light incident to both sides of the head was held either (i) ‘horizontally' or (ii) ‘vertically' by rotating the filter 90°; so the horizontally or vertically polarized light was transmitted at low elevations, respectively, creating two orthogonal polarization patterns around the larvae. After application, the larvae proceeded under (iii) unobstructed sky without a filter. The PF created shade (transmission to unpolarized light 40%) and additionally decreased the incident light by maximum 30%, depending on the angle with respect to the polarized skylight [21]. As the stemmata are non-image-forming organs with large fields of view, the possible intensity artefact slightly affected the total, but not the differential signal in the orthogonal photoreceptor pairs. Thus, (iv) control experiments were performed using a 45% neutral density filter (NDF) (Lee 298 and 209 combined; Lee Filters, Hampshire, UK) in place of PF. Each treatment lasted for 2 min, and the larval orientation was recorded 20 s after commencement (figure 1). The larvae were changed after every trial.

The same experiments were conducted on final 8th instar (L8) *O. lunifer* (*N* = 12 processions) and 5th instar (L5) *T. pityocampa* (*N* = 7 processions) larvae in their natural habitat without the arena. *Ochrogaster lunifer* were observed in late March 2019 at 06.00–12.00 h (GMT + 10) at The University of Queensland, Gatton campus, Queensland, Australia (−27°56′ S, 152°34′ E). Summer feeding *T. pityocampa* [22] were observed in September 2019 at Leiria, Portugal (39°31′ N, 9°07′ W) at 08.00–13.00 h (GMT + 1).

### (b) Morphological analyses

Preparation of stemmata for SEM, LM and TEM was performed as described previously [23]. Details can be found online [24].

### (c) Statistical analyses

All analyses were performed using R Studio version 1.1.419 and an alpha value of *p* < 0.05 was taken as statistically significant. In some trials, the PF was applied and removed repeatedly on the same procession/singleton. Before pooling the data for analyses, we tested if the orientation of processions was affected by previous exposure to the PF. The Wallraff Test of Angular Distances was performed using the RStudio software package ‘circular' [25] on all processions/singletons. There were no significant differences (all *p* > 0.1); therefore, the data were used as independent for each treatment. To determine if the procession/singleton reacted to the treatments, the angular difference was calculated by subtracting the initial orientation of travel from the manipulated orientation after PF/NDF exposure/removal. Summarized angular differences of each group after the treatments were graphed as circular plots with a kernel density estimation (KDE; figure 1*g*–*i*). The angular difference was given a score of 0 or 1 according to the difference being less (no change) or greater than 22.5° (change; 22.5° is the minimal cardinal value on the compass). Circular logistics regression model (CLRM) for binomial data [26] was applied on the angular difference using the variables: treatment, azimuth angle (degrees), procession/singleton ID, number of larvae in the procession and starting orientation. The models were reduced to the model of best fit by removing non-significant covariables and by lowering the Akaike's information criterion value. Environmental temperatures of the study sites were collected, but not used as a variable because of collinearity (*r* > 0.70) with the Azimuth angle. Details of R codes used can be found online [24].

## 3. Results and discussion

### (a) Behavioural analyses

Application of PF either ‘horizontally' or ‘vertically' resulted in a directional change of the leader and the rest of the procession by 58–103° (table 1), creating a zig-zag column (figure 1*a*–*f*). However, the PF attenuated the incident light. We tested for this effect by using a NDF, which caused a minor heading change of 18–32° (table 1). None of the other explanatory variables contributed to the angular difference (all *p* > 0.1) for all three groups: L1 and L5 *T. pityocampa* and L8 *O. lunifer*. The angular differences for NDF were significantly different from all other treatments in *T. pityocampa* L1 and for PF horizontal in *O. lunifer* L8 (table 2; electronic supplementary material, figure S2); NDF produced minimal deviation in orientation. For *T. pityocampa* L5, the NDF was unavailable; therefore, the next variable in alphabetical order, PF horizontal, was compared against the angular difference of PF vertical and removal. Angular differences between the treatments were not statistically significant, suggesting that any PF angle produced a change in larval orientation. Our results support findings [12,13,27] that holometabolous larvae change their orientation of travel proportionally to the PF angle. The polarized pattern alone is an ambiguous cue and its rotation by 90° should lead to directional changes by −90° or +90°. The zig-zag path of the procession indicates that the directional changes were roughly consistent between trials with and without PF (including NDF). Thus, the caterpillars were probably orienting using additional cues such as the solar position or landmarks. Skylight polarization cues may be particularly important for processionary caterpillars because individuals separated from processions could re-join their colony at pupation or nesting sites. Being gregarious is beneficial for both species because the increase in group size can increase larval survival through cooperative defence strategies and by dilution effects [28,29]. Doane and Leonard [13] found that after PF removal, larvae returned to their original direction. In our study, this was observed in 21% of the trials, suggesting that most larvae set a new heading orientation after each PF treatment, similar to e.g. tethered fruit flies, flying below PF [30,31].

**Table 1.** Mean ± standard error of the angular difference (degrees) after exposure to each treatment.

groups	NDF	PF horizontal	PF vertical	PF removal	*N*
*Thaumetopoea pityocampa* L1	32 ± 10	102 ± 6	62 ± 11	96 ± 10	113
*Thaumetopoea pityocampa* L5	—	59 ± 15	101^a^	72 ± 6	15
*Ochrogaster lunifer* L8	18 ± 18	103 ± 17	58 ± 58	49 ± 22	26
N	34	55	31	34	

— data unavailable.

^a^SE was not calculated, not enough data.

**Table 2.** Results from circular logistics regression model for binomial data. Binomial data from the angular difference of NDF was compared against other treatments.

groups	PF horizontal		PF vertical		PF removal		*N*
	*z*	*p*	*z*	*p*	*z*	*p*	
*Thaumetopoea pityocampa* L1	3.99	<0.001	2.62	0.009	3.89	<0.001	113
*Thaumetopoea pityocampa* L5^a^	—	—	0	1	0.001	1	15
*Ochrogaster lunifer* L8	2.74	0.006	0.003	1	0.006	1	26

— data unavailable.

^a^NDF data unavailable, PF horizontal was compared against other treatments.

### (b) Morphological analyses

**Figure 2.**
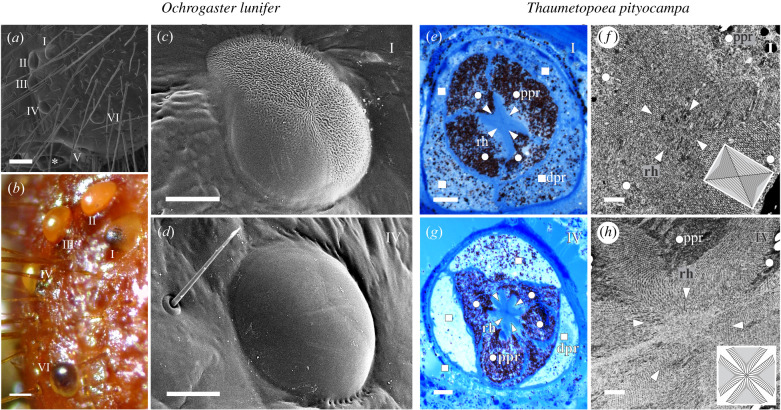
Anatomy of *O. lunifer* (*a–d*) and *T. pityocampa* (*e–h*) stemmata. (*a*,*b*) Side view of the left head capsule of final instar larva; (*a*) scanning electron micrograph (SEM); (*b*) stereomicrograph, stemma V hidden. Numbers refer to stemmata I–VI. (*c*,*e*,*f* ) Stemma I; (*c*) SEM of the cornea; (*e*) light micrograph (LM) of the photoreceptors; (*f*) transmission electron micrograph (TEM) of the rhabdom. (*d*,*g*,*h*) Stemma IV; (*d*) SEM of the cornea; (*g*) LM of the photoreceptors; (*h*) TEM of the rhabdom. The bottom right corners in (*f*) and (*h*) are schematic diagrams of the rhabdom illustrating the microvillar orientation. In (a), the antenna is indicated by an asterisk; in (*e,f,g,h*), rhabdomeres (rh) are indicated by triangles, proximal photoreceptor bodies (ppr) by circles and distal photoreceptor bodies (dpr) by squares. Scale bars: (*a*) 200 µm; (*b*) 100 µm; (*c*,*d*) 50 µm; (*e,g*) 10 µm; (*f,h*) 1 µm.

Larvae of both species have six stemmata on each side of the head (figure 2*a*). Stemmata II–VI appear shiny, while stemma I appears opaque (figure 2*b*). SEM revealed that 2/3 of the surface of stemma I pointing upwards is rugged and 1/3 pointing downwards is smooth (figure 2*c*), whereas stemmata II–VI are smooth all around (figure 2*d* and electronic supplementary material, figure S3). The dark pigment in the lower third indicates that stemma I samples the dorsal part of the visual field (figure 2*b*). The rugged surface of stemma I is a diffuser and a spatial low-pass filter for incident light. A similar opaque optical structure has evolved in the DRA ommatidia of honeybees and locusts [7,32]. These structures enlarge visual fields, decrease acuity and are thought to reduce the visual clutter caused by clouds, thereby stabilizing skylight polarization vision [7]. We hypothesized that stemma I could harbour photoreceptors optimized for polarization vision. Indeed, LM and TEM images showed a single-tiered, cushion-shaped light-sensitive structure (rhabdom), formed of two pairs of photoreceptors with orthogonal and aligned microvilli (figure 2*e*,*f*; electronic supplementary material, figures S4 and S5). Other stemmata have flower-shaped rhabdoms, formed of more than 3 photoreceptors in the distal and proximal tier; their microvilli are neither aligned nor orthogonal (figure 2*g*,*h*; electronic supplementary material, figures S4 and S5). The rhabdom in stemma I is similar to rhabdoms in DRA ommatidia of Noctuid and Crambid moths, while the rhabdoms in stemmata II–VI resemble the lobed rhabdoms in the main retina of adult moths [23,33]. The conspicuous dioptrical apparatus and rhabdom clearly indicate that stemma I is optimized for polarization vision, while other stemmata do not show such optimizations and are thus suitable for general vision, such as intensity or colour contrast detection.

## 4. Conclusion

We have demonstrated that stemma I in social caterpillars is anatomically similar to a single ommatidium in the DRA of adult moths. Although it is a non-imaging polarization detector that samples only a fraction of the skylight pattern, a pair of stemmata is still capable of assisting spatial orientation, similar to the specialized simple eyes in certain spiders [34]. Larval locomotion was manipulated with a PF, which showed that polarization vision is one of the mechanisms that guided their social behaviour. Skylight polarization pattern enables pre-pupation processions to have a constant heading away from the nest to disperse further and to avoid drift, which could result in a loop. Stemmata with a rugged surface are absent in solitary caterpillar species studied to date [35,36]. It will be interesting to investigate if this trait is conserved in Notodontidae or evolved independently in different families. Social behaviour and organized locomotion exert strong selective pressure on the visual organs, which in turn robustly convey visually guided behaviour, even in the simplest structural form.

## Data accessibility

Histological methodologies and R codes for statistical analyses are provided in Dryad dataset link: https://doi.org/10.5061/dryad.9w0vt4bdh.
